# Direct Observation of Coherent Oscillations in Solution due to Microheterogeneous Environment

**DOI:** 10.1038/srep06097

**Published:** 2014-08-18

**Authors:** Dipak Kumar Das, Krishnandu Makhal, Soumendra Nath Bandyopadhyay, Debabrata Goswami

**Affiliations:** 1 Department of Chemistry, Indian Institute of Technology Kanpur, Kanpur-208016, Uttar Pradesh, India

## Abstract

We report, for the first time, direct observation of coherent oscillations in the ground-state of IR775 dye due to microheterogeneous environment. Using ultrafast near-infrared degenerate pump-probe technique centered at 800 nm, we present the dynamics of IR775 in a binary mixture of methanol and chloroform at ultra-short time resolution of 30 fs. The dynamics of the dye in binary mixtures, in a time-scale of a few fs to ~740 ps, strongly varies as a function of solvent composition (volume fraction). Multi-oscillation behavior of the coherent vibration was observed, which increased with decreasing percentage of methanol in the dye mixture. Maximum number of damped oscillations were observed in 20% methanol. The observed vibrational wavepacket motion in the ground-state is periodic in nature. We needed two cosine functions to fit the coherent oscillation data as two different solvents were used. Dynamics of the dye molecule in binary mixtures can be explained by wavepacket motion in the ground potential energy surface. More is the confinement of the dye molecule in binary mixtures, more is the number of damped oscillations. The vibrational cooling time, τ_2_, increases with increase in the confinement of the system. The observed wavepacket oscillations in ground-state dynamics continued until 1.6 ps.

Microheterogeneous environment for a dye molecule can be generated by mixing two miscible solvents in certain proportions^1^. It plays an important role in physicochemical and biological processes^2^,^3^. Various photophysical properties of molecules or dyes are strongly affected in microheterogeneous enviroments^4^,^5^,^6^. Characterization of microheterogeneous environment and understanding its impact on the solute molecules is still under progress. The dynamical processes, in particular, occurring due to microheterogeneous environment, are unclear even in the recent decade^7^. Lately, binary mixtures of solvents have drawn attention from the scientific community as microheterogeneous environment because of their diverse applications as cryo-protecting agents^8^, low temperature glasses^9^ as well as for observing the antagonistic effect on a protein structure^10^. Recent studies have shown that the microheterogeneous environment of the binary mixture of (MeOH) and chloroform (CHCl_3_) can strongly affect fluorescence quantum yields, *trans-cis* isomerization and fluorescence lifetimes^7^.

Binary mixtures of methanol (MeOH) and chloroform (CHCl_3_) are important because of their complete miscibility in all ratios^11^. The high solubility of lipophilic and amphiphilic molecules in the binary mixtures of MeOH-CHCl_3_, is important and advantageous in their synthesis, chromatography and spectroscopy^11^. This mixture is widely used as a function of isotropic membrane mimics in high-resolution NMR spectroscopy of membrane bound compounds^12^,^13^,^14^,^15^. Another important advantage of this mixture lies in the formation of homomolecular or heteromolecular clustering^16^,^17^. For better understanding of this structure (*i.e.*, clustering) and the properties of MeOH-CHCl_3_ binary mixture, empirical force field methods, such as Molecular Dynamics (MD) or Monte Carlo (MC) methods, are mainly used for theoretical calculations and these results are in agreement with the experimental data^11^,^18^.

The coherent vibrational motion of a large polyatomic dye molecule in solution was investigated for the first time in 1986 by Tang's group, using femtosecond transmission correlation technique^19^. They demonstrated that interferences between coherently excited vibrational levels resulted in the observed beat frequencies, which correspond to the energy spacing between the vibrational levels^19^,^20^. The pump-probe measurements using very short femtosecond pulses were able to determine the underlying vibrational frequencies of the dye molecule in solution^21^. Yang *et. al.* studied the molecular structure and dynamics of a cyanine dye in solution by using femtosecond pump-probe technique with a time resolution of 90 fs at the red edge of the absorption band^22^. They correlated their experimental results with the support of theoretical calculations using 11 fs laser pulses. The coherent motion of a cyanine dye molecule was also observed by using chirped pulses^23^. The coherent motion in the excited state depends on the sign of the chirp for the pump pulse. The positively chirped pulses transferred a large amount of population to the excited state, whereas the negatively chirped pulses, just after excitation by the pump pulse, almost completely dumped the excited state population^23^,^24^. Investigations of coherent motion of a dye molecule with 6 fs pulses were carried out by Shank group^25^. The dampening of the coherent vibrational motion occurs at a much shorter timescale in the solution phase as compared to that in gas phase due to solvent solute interactions.

When a solute molecule releases excess vibrational energy to the surrounding solvent molecules in order to achieve thermal equilibrium, it is popularly called vibrational cooling^26^,^27^,^28^,^29^,^30^. Heat dissipation initially occurs from the solute molecule to the first solvation shell and then it gets transferred to other solvent shells, which are closer to the first solvation shell. Heat dissipation of a solute molecule mainly depends on its size (number of atoms present in the molecule) and properties of the solvent(s) (*e.g.*, density, viscosity, refractive index, polarity, etc.)^27^. Vibrational cooling rate is higher for hydrogen binding solvents and lower for unassociated organic liquids. Very recently, it has been confirmed that vibrational cooling also depends on the isotopes of an atom for a given solvent (e.g. H_2_O and D_2_O) and increases with increase in the atomic number of the isotope^29^. The excited state life time for vibrational cooling is larger than the Internal Conversion (IC) process for a molecule in the excited state and it varies from timescales of sub-picosecond to picoseconds^30^.

Dyes or molecules that are active in the Near-IR (NIR) region (650–900 nm) have been in focus since past decade because of their diverse applications in Pharmacology, Molecular and Cellular Biology, Diagnostics and Material Science^31^. The NIR dyes are also important in bio-imaging (in-vivo/in-vitro) due to low toxicity, less harmfulness and very low background signal^32^. They are mainly used as a single photon dye due to their high absorption cross-sections in the NIR region^33^. Cyanine dye is the most important NIR dye, and is readily available^31^,^34^.

In this paper, we present the effect of microheterogeneous environment on the ground state dynamics of IR775 dye in a binary mixture of MeOH-CHCl_3_ by using degenerate pump-probe technique with a resolution of 30 fs. To the best of our knowledge, this is the first report on the direct observation of vibrational wavepacket in the ground state of a dye molecule due to microheterogeneous environment. We clearly explain the reason behind the coherent oscillations and the increase of the number of oscillations in the ground state due to microheterogeneous environment.

## Methods

### Materials

**Figure 1:**
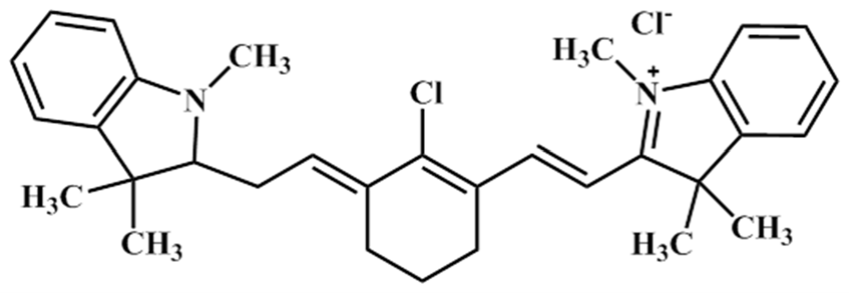
Dye structure of IR775.

We purchased commercially available NIR dye (2-[2-[2-Chloro-3-[2-(1,3-dihydro-1,3,3-trimethyl-2H-indol-2-ylidene)-ethylidene]-1-cyclohexen-1-yl]-ethenyl]-1,3,3-trimethyl-3H-indolium chloride), commonly known as IR775 from Sigma-Aldrich (USA, Inc.) and used it as received. Molecular structure of the dye is shown in Figure 1. Spectroscopic grade MeOH and CHCl_3_ were purchased from Rankem, India. They were used without further purification. All the binary mixtures with IR775 dye were prepared by mixing these two solvents volume wise. We kept the solutions of IR775 in the binary mixture, in a cool and dark place before our steady-state and time-resolved study. All experimental data were recorded at room temperature (295 K).

## Experimental Methods

### Steady State Measurements

#### (a) UV-Vis-NIR absorption

The steady state absorption spectra of IR775 in MeOH-CHCl_3_ binary mixtures were measured with a UV-Vis-NIR *Absorption Spectrophotometer* (JASCO, V670) at 0.2 nm resolution, using 1 mm optical path-length quartz sample cell (Hellma, USA). In order to avoid dye aggregation, concentrations of all the solutions were kept at ~10^−5^ M. We collected the absorption spectra after the laser irradiation and noticed no significant changes in the respective absorption spectra. This indicates absence of photo-degradation during our pump-probe experiments.

#### (b) Fluorescence Measurements

Fluorescence spectra of the individual dye mixtures were recorded by using a home-built experimental setup. We used the amplified laser pulse as the source of excitation, which was also used for time-resolved measurements. The laser pulses were then focused with a 5 cm focal length lens onto a 10 mm quartz sample cell (Hellma, USA) filled with the sample solution. A microscopic objective (N.A. ~0.3) was used for collecting the fluorescence onto a fiber coupled miniature Grating Spectrometer (Ocean Optics, 2000 USA Inc.), which was interfaced with a personal computer. The experimental data recorded for both absorption and fluorescence were plotted using Origin 8.5 program.

### Time-resolved measurements

Transient absorption measurements were performed by using the conventional pump-probe technique. The laser system used in our experiments is a commercially available Mode-locked Regenerative Amplified Laser (Spitfire Pro, Spectra Physics, USA, Inc.), which generates near Transform-limited (TL) ~30 fs laser pulses at 1 kHz repetition rate having 800 nm central wavelength and 4 mJ/pulse peak power. Pulse width of the amplifier was measured by intensity autocorrelation technique using 0.01 mm thin type-I BBO crystal (Castech, θ = 30°, Φ = 33°). Commercially available Mai-tai oscillator, which generates 20 fs (Full Width at Half Maximum (FWHM) ~60 nm) pulses with ~8 nJ pulse energy and repetition rate of 80 MHz, is used as a seed laser to feed the amplifier. A nanosecond Q-switched Nd:YLF laser system (Empower, spectra physics, USA, Inc.), which generates laser pulses of 527 nm central wavelength at 1 kHz repetition rate with 19 mJ pulse energy, is used to pump the amplified laser system. The amplified laser pulses were then divided into two equal halves by using a 50:50 ultrafast thin *Beam-Splitter* (BS). The reflected beam from the front surface of the BS was used as the pump beam. The transmitted part of the laser beam, which travel through the retro reflector is used as the probe beam. The retro reflector was mounted on a computer controlled, stepper motor driven, translation stage (UTM 150 PP.1, Newport, USA) having a resolution of 0.1 μm. The transmitted beam, after it had gone through a variable delay line, was used as a probe beam. Data collection and stepper motor positioning were controlled by in-house LabVIEW 8.5 program that ran on a personal computer, interfaced with GPIB. The pump and the probe beam were combined non-collinearly by using a 10 cm focal length lens. The angle between the pump and probe pulse at focus was kept at ~4°. The zero delay of the optical path was measured by putting a thin type-I BBO crystal at the focal point of the two beams. The pump power was 100 times higher than that of the probe beam and the beam diameter of the pump beam was kept smaller than that of the probe beam. This was done to maintain the 2:1 ratio of the pump and probe diameter at the focus. The sample solution was placed in a 1 mm cuvette at the focus of the two laser beams which also correspond to their overlap region. The relative transmittance of the probe beam was measured as a function of its delay with respect to the pump beam. The probe transmittance was collected on a fast photodiode (DET210, Thorlabs) connected to a digital oscilloscope (LeCroy 64Xi), which in turn was interfaced with a personal computer. The relative transmittances for various delays were plotted with the MATLAB® program.

## Results and Discussion

### Steady State Absorption and Emission Spectra of IR775 in the Binary Mixtures of Methanol and Chloroform

**Figure 2:**
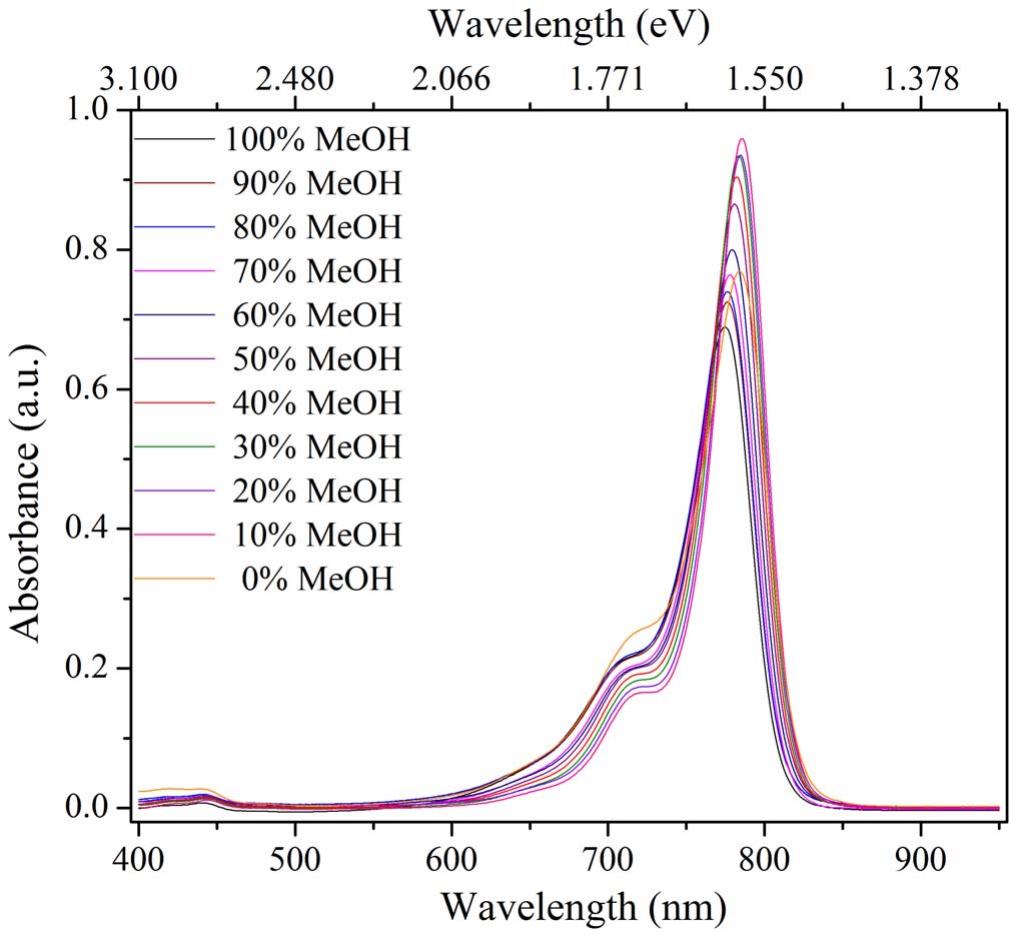
Absorption spectra of IR775 in the binary mixture of MeOH-CHCl_3_ at different volume ratios.

**Figure 3:**
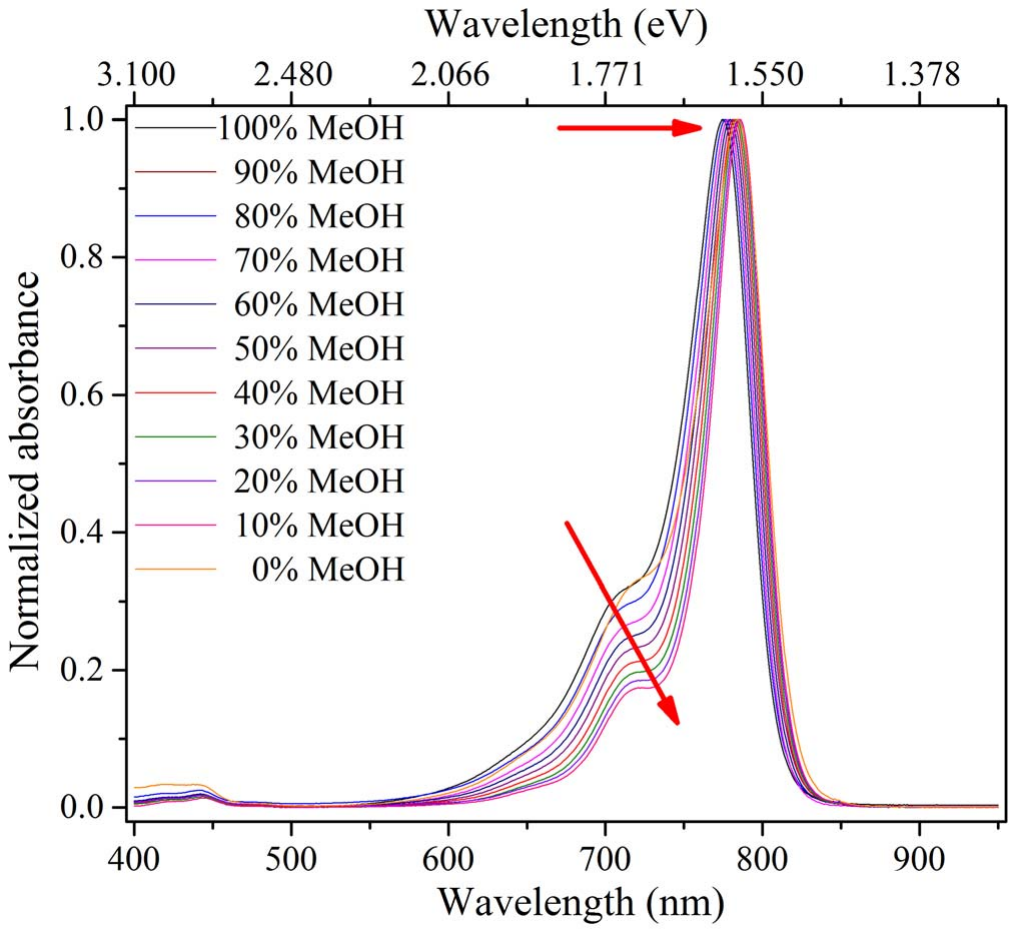
Normalized absorption spectra of IR775 in the binary mixture of MeOH-CHCl_3_ for different volume ratios.

The steady state absorption spectra of IR775 in eleven different binary mixtures of MeOH-CHCl_3_ from 100:0 down to 0:100 are shown in Figure 2. The absorption band located in the NIR region with its peak ranging 775–785 nm is attributed to the *S*_1_ ← *S*_0_ transition under different compositions of the binary mixtures of the IR775 dye. We observed that the peak positions of the absorption band were shifted to lower energy region (more red shifted) over ~164 cm^−1^ and the FWHM of the absorption band also increased over 194 cm^−1^ with decreasing methanol content in the binary mixtures. The maximum red shift in the absorption spectra was recorded in 100% CHCl_3_. The vibronic side band on the higher energy side increased with increasing methanol content in the binary mixtures and is shown in Figure 3. This indicates that the ground state energy changes with changing compositions of the binary mixture. We observed a small change of the vibronic side band over ~215 cm^−1^ in the higher energy side with decreasing methanol content. The assignment of a very small absorption band in the visible region (see Figure 3) is attributed to *S_n_* ← *S*_0_ (n ≥ 2) transition. We noticed that there were no significant changes in the absorption peak wavelength in the visible region.

**Figure 4:**
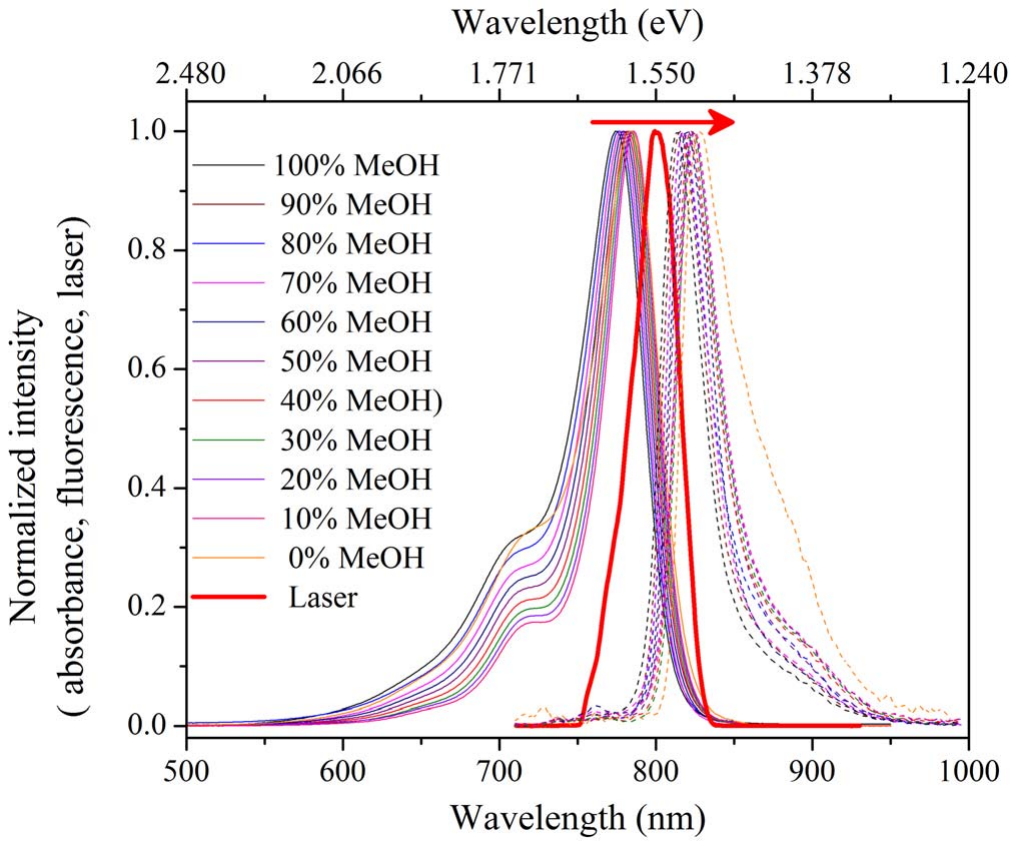
Normalized absorption (solid line), fluorescence (dotted line) spectra of IR775 in MeOH-CHCl_3_ binary mixture (for each absorption and fluorescence, same color code is used). For comparison, spectrum of the amplified femtosecond laser is shown in thick red line.

Figure 4 shows the laser spectrum, steady state absorption spectra and emission spectra of IR775 in the binary mixture of MeOH-CHCl_3_. We observe a lack of mirror image relationship between the absorption and emission spectra of our dye molecule in the binary mixtures. This indicates that the ground and excited state energy spacings are not the same due to geometrical relaxation of IR775 dye after photo-excitation. We also observe that the peak wavelength of the emission spectra shifts to the lower energy side with decreasing methanol content in the binary mixture and the maximum red shift was observed in 0% methanol (*i.e.*, 100% CHCl_3_).

**Figure 5:**
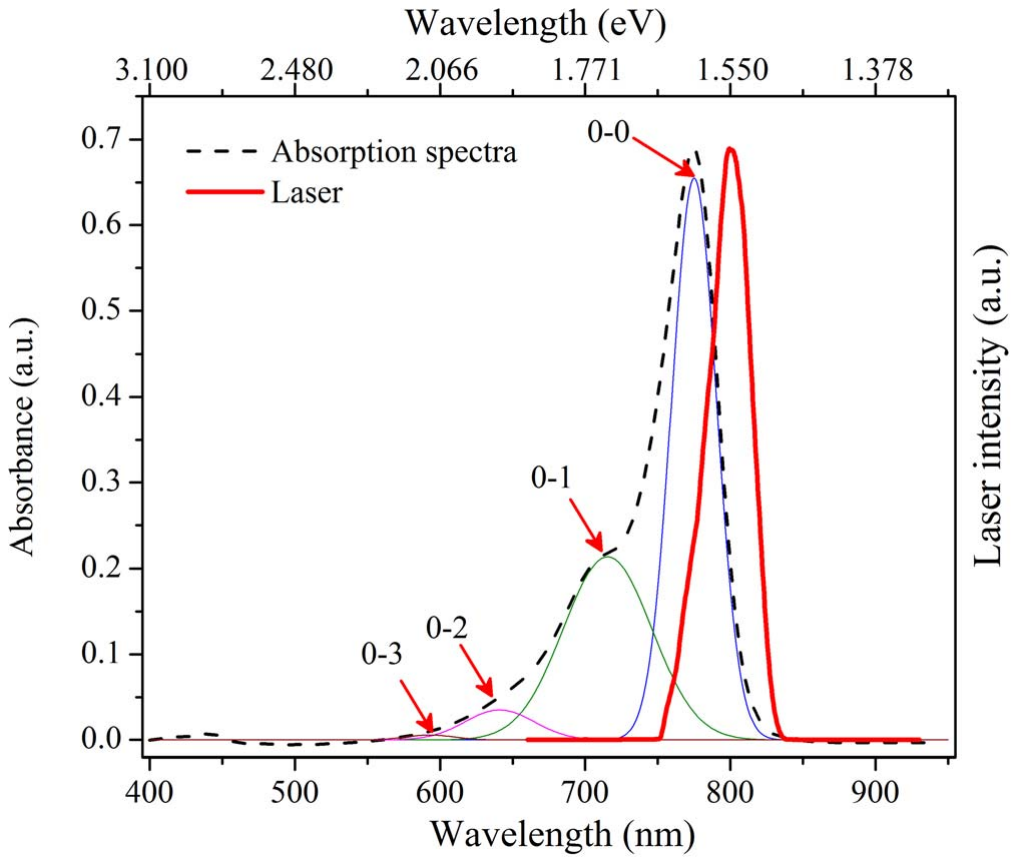
Absorption spectrum of IR775 in 100% methanol (black dotted line) and the amplified laser spectrum (red thick line). The thin lines are the Gaussian deconvolution plots showing the transitions from the zeroth vibrational level in the ground state to the *i*^th^ (where i = 0, 1, 2, 3) vibrational level in the excited state.

Figure 5 shows that the stationary absorption spectrum for IR775 in 100% methanol can be fitted with a set of four Gaussian bands due to the transition from the ground vibrational state (v = 0) to the four possible excited vibrational states (v′ = 0, 1, 2 and 3). Similar deconvolution fitting can be performed for all the absorption spectra of our IR775 dye in different binary mixture concentrations by considering the transitions from the zeroth vibrational level in the ground state to the *i*^th^ (where *i* = 0, 1, 2, 3) vibrational level in the excited state corresponding to the vibrational quantum numbers v′ = 0, 1, 2 and 3 in the excited state (all deconvolution plots are shown in supporting information Figure S1). However, our laser spectrum covers only the v′ = 0 and 1 excited vibrational states (see figure 5).

We measure the peak shifts of both the absorption and fluorescence maxima from the absorption and fluorescence spectra of IR775 in MeOH-CHCl_3_ binary mixtures. The peak shifts in the absorption and fluorescence maxima are indicative of shifts in both the ground (S_0_) and the first excited electronic energy states (S_1_). Such ground and excited state potential energy shifting indicate that there occurs a displacement of the ground and excited state potential energy surfaces, which is denoted by Δ and the value of 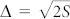 where S is known as the Huang-Rhys Factor. These shifts in the ground and excited state potential energy surfaces are schematically shown in figure S2 (in the supporting information).

### Transient absorption experiment of IR775 in binary mixtures of methanol and chloroform

**Figure 6:**
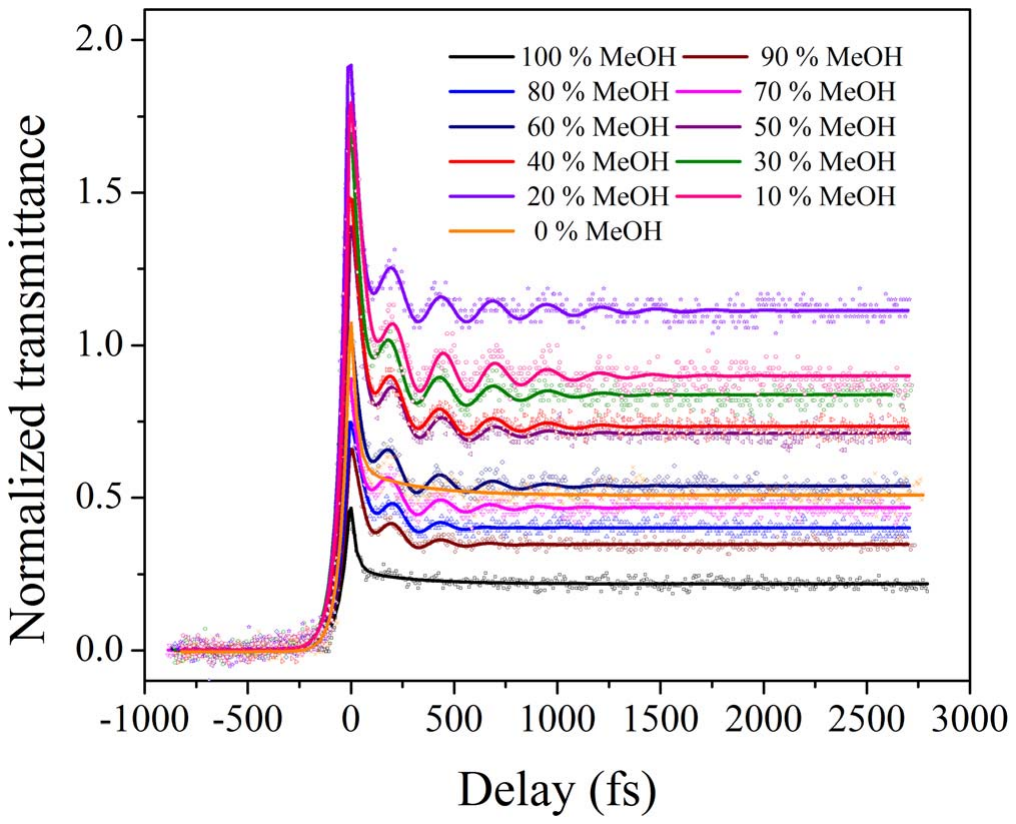
Initial decay of the transient absorption of IR775 in the binary mixture of MeOH-CHCl_3_.

The degenerate transient absorption of IR775 dye in a binary mixture of MeOH-CHCl_3_ was recorded from an extreme time resolution of 30 fs to 740 ps. First, the initial decay time, ranging from 30 fs to 2.7 ps, for all our samples (IR775 in varying binary mixture solutions) were analyzed and is shown in Figure 6. We visualize a coherent spike or coherent artifact at zero delay which reflects the characteristics of the laser pulse. To remove the effect of coherent spike in the dynamics, we fitted the data after 30 fs. We observe that the intensity of the transmittance increases with a decrease in the methanol content in the binary series and the maximum transmittance was recorded for 20% beyond which, at 10% it again decreases. We also observed a coherent oscillation in the initial decay of the transient absorption, which increases with a decrease in the methanol content in our binary series.

**Figure 7:**
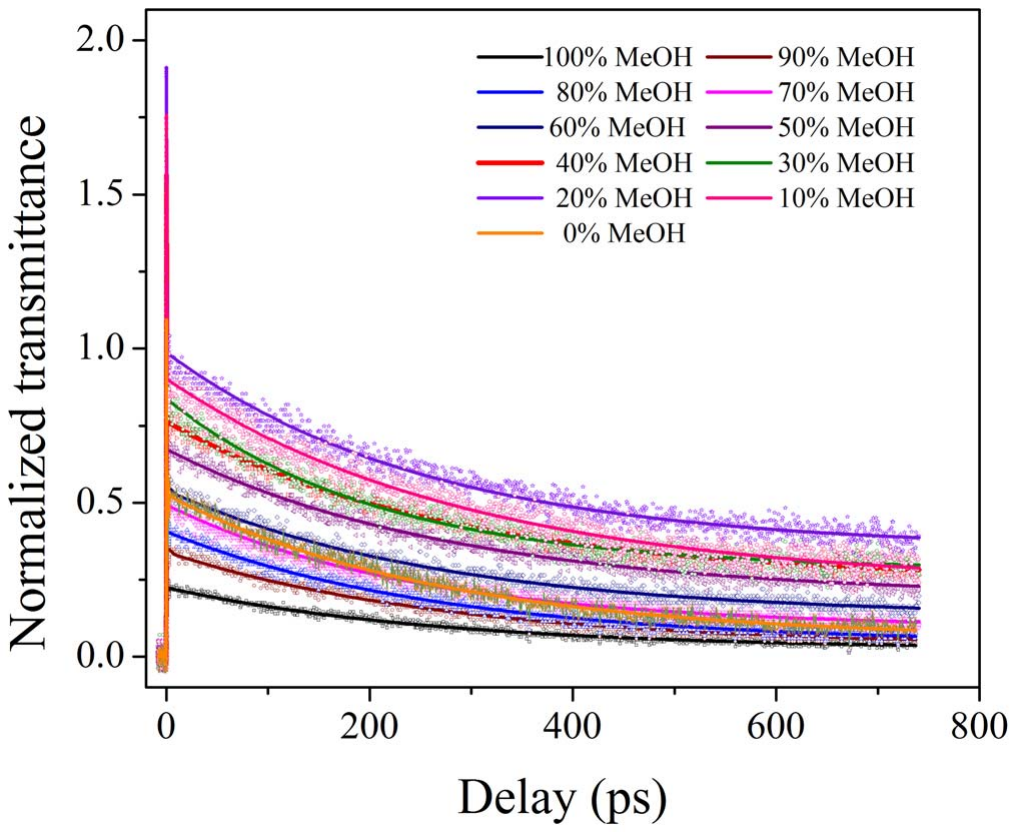
Full decay of the transient absorption of IR775 in the binary mixture of MeOH-CHCl_3_.

In a standard pump-probe experiment, the coherent oscillations are generated in both the ground and excited states. However, earlier experiments^22^ have shown that in the particular case of degenerate pump-probe experiments, the ground state contribution increases as the excitation wavelength shifts towards the redder side of absorption maxima, and the observed coherent oscillations between the ground and excited state disappear. Our degenerate pump-probe experiments with 800 nm has redder excitation wavelength as compared to our dye's absorption maxima that range from 775–785 nm (depending on the composition of dye in the binary mixtures of methanol and chloroform). The very existence of the coherent oscillations indicate a coherence between the ground and excited states and a finite contribution of both the states. We expect our coherent oscillations to have a major contribution from the ground state under our red-shifted experimental conditions. Since the absorption spectrum of the dye molecule blue-shifts as the methanol concentration increases in the binary mixture, the coherent oscillations appear and become stronger as the dye's absorption spectrum in the binary mixture moves closer towards the laser spectrum. Thus the maximum number of coherent oscillations in our experiment was recorded in 20% methanol. The absence of coherent oscillation in the initial decay of neat-MeOH and neat-CHCl_3_ solution (see Figure 6) indicates that the coherent oscillation generated in the ground state dynamics is a result of microheterogeneous environment over and above the spectral shift near resonance. Figure 7 shows the full decay dynamics of IR775 dye in MeOH-CHCl_3_ binary mixtures.

In our experiments we performed the degenerate pump-probe experiments for observing the coherent oscillations of IR775 in binary mixtures of MeOH-CHCl_3_ at room temperature (295 K). We do not perform any temperature dependent time resolved study. This is because, an attempt to perform temperature dependent study of such a mixture would also result in an implicit change in the viscosity of the binary mixtures. Consequently, the environment of the dye in binary mixtures will be different with temperature variation. One of the reasons for the choice of methanol and chloroform binary mixture has been the fact that at room temperature their viscosities are almost same (viscosity of methanol = 0.593 cP at 20°C and viscosity of chloroform = 0.563 cP at 20°C) and as such the binary mixture environment is unchanged across their relative composition variations as presented in our study. Temperature dependent study will end up producing different environment in our experiments, which will not allow us to present the trend as we have demonstrated in the current study.

In addition to the central wavelength of the laser, laser pulse width also plays an important role in observing the coherent oscillations (ground/excited state) for molecular systems. In our current experiments we have observed coherent oscillations using 30 fs pulses, which disappear with an increase in the laser pulse width. This indicates that in order to observe the coherent oscillations, extremely short laser pulses (~30 fs) are necessary that can cover a large frequency space thereby enabling coherence between them. We needed two cosine functions to appropriately fit the coherent oscillation behavior of the dye in the binary solvent mixtures as the relative composition of the two different solvents in the binary mixtures were responsible for nature of the oscillations. From the fitted data we observed that the periodicity of the oscillations vary with the composition of the binary mixtures and is shown in Table 1.

We correlate our present experimental results to the theoretical calculations which were done previously^18^. From Figure 6, we find that with decreasing volumes of MeOH in MeOH-CHCl_3_ binary mixture, the probability of formation of cluster becomes higher and higher to finally reach the maximum at 20% MeOH. This indicates that the coherent oscillations, which in our particular case, are primarily indicative of the ground state dynamics, depend on the environment around the IR775 dye molecule(s). As reported previously, MeOH-CHCl_3_ binary system mainly exhibits cluster formation, depending on the composition of the two individual solvents which was explained theoretically using MD or MC calculations^18^. The theoretically simulated data were found to be in good agreement with the earlier experimental results^11^. We can argue that, with more cluster formation, the system becomes more and more stable due to confinement. In our experiments, we get the maximum number of coherent oscillations of the dye at 20% MeOH, which again decreases at 10% MeOH. This indicates that the system becomes most confined due to cluster formation around the dye molecules resulting in the maximum number of oscillations at 20% MeOH in MeOH-CHCl_3_ binary mixture.

The oscillatory trace of Figure 6 was fitted to a superposition of two cosine functions with different amplitudes (A_1_, A_2_), phases (*ϕ*_1_, *ϕ*_2_) and periods (T_1_ and T_2_) multiplied by an exponential decay function. We used linear prediction singular value decomposition (LPSDV) technique to fit our time-resolved experimental data using the following equation: 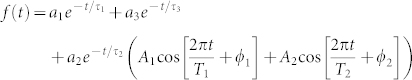


The fitted equation contains the amplitudes *a*_1_, *a*_2_ and *a*_3_, of which *a*_1_ and *a*_3_ are the two pre-exponential factors. The first decay τ_1_ gives the time constant corresponding to the Intramolecular Vibrational Energy Redistribution (IVR). The second decay time constant τ_2_ gives the Vibrational Cooling time (VC) and the long decay constant τ_3_ indicates the ground state recovery time.

Table 1 shows that *τ*_2_ > *τ*_1_ for our experiments, which is in good correlation with the previously reported results for various dyes in different solvents^30^. We also observe from table 1 that the τ_2_ values of our MeOH-CHCl_3_ binary mixture increased on decreasing the volume of MeOH and it became maximum at 20% MeOH. This reaffirms the fact that on decreasing the volume of MeOH for the corresponding increase in CHCl_3_ volume in the MeOH-CHCl_3_ binary mixture, more and more confinement of the dye molecule(s) occur due to cluster formation of MeOH-CHCl_3_ around the dye molecule(s). This makes heat dissipation of the dye molecule into the bulk solvent more time consuming. This result is also in good correlation with the solvent dependent VC, as recently reported^28^. The maximum error bar for fitting our experimental data is ~7% from residual plots.

We observe from Figure 6, that the phase of coherent oscillations due to the vibrational wavepacket remains the same under different compositions of our MeOH-CHCl_3_ binary mixtures. The amplitude of the coherent oscillations increases monotonically from 90% methanol to 20% methanol in the MeOH-CHCl_3_ binary mixtures. The maximum amplitude of oscillation is at 20% methanol. Thereafter the oscillation amplitude decreases for the 10% MeOH case. All these information are summarized in Table 1. The oscillation time period, T_1_ and T_2_ slightly changes depending on the binary mixture compositions of the dye as shown in table 1.

In our experiments, the vibrational oscillations are generated due to the following reason. Either in neat-MeOH or in neat-CHCl_3_ solution, the probability of cluster formation around the dye molecule is very little. This implies that no such vibrational wavepacket generation is possible in the ground state potential. Under microheterogeneous environment, however, the systems become more confined due to cluster formation. The ground state vibrational wavepacket moves along the ground state potential energy surface. We observe coherent oscillations when the frequency of the wavepacket matches with the energy difference or energy spacing between the two successive vibrational levels (here 0–0 and 0–1 state of S_0_ electronic state). In our MeOH-CHCl_3_ binary mixtures, the probability of oscillation increases with increasing cluster formation, and it becomes maximum for 20% MeOH.

We have performed our degenerate pump-probe experiments of IR775 in MeOH-CHCl_3_ binary mixtures with eleven samples each varying at 10% volume increment of MeOH. If we try to perform more experiments at intervals that are smaller than 10% increments in MeOH concentration in CHCl_3_, we confront the volume error bar limits of the binary mixture. The error bars come from the fact that the mixing process of Methanol with Chloroform to form the binary mixtures show a negative volume mixing^35^ (ΔV_mix_ = -ve) over the entire range of volume-fraction that we are studying. Error due to this volume mixing is in the range of 5%, which precludes our capability to measure any reliable binary concentrations in increments less than 10%. Given our measurement limit, when we count the number of coherent oscillations, we find that the maximum oscillation are observed at 20% MeOH (8 oscillations), while the next high are for 30% and 10% MeOH cases, respectively (each having 6 oscillations).

We observed from table 1 that, in our binary series, the longtime decay constant, *τ*_3_ ≥ 245 ps depends on the composition of MeOH-CHCl_3_. In our experiments, *τ*_3_ becomes minimum in neat-MeOH and neat-CHCl3 and increases on changing the composition of the binary solvents. This experimental result gives the important information that *long time decay constant* (τ_3_) is mainly dependent on the microheterogeneous environment.

## Conclusion

We carried out degenerate pump-probe experiments with 30 fs time resolution using NIR pulses at 800 nm to measure the effect of microheterogeneous environment on the vibrational dynamics of IR775 in varying binary mixture compositions of MeOH and CHCl_3_. Coherent oscillation in the ground state increases with decreasing MeOH content in the binary mixtures to reach the maximum at 20% MeOH. As we decrease the MeOH concentration and increase the CHCl_3_ content in the binary mixtures, formation of binary clusters occur, which makes the system more and more confined. With increasing confinement, the dynamics of the ground state wavepacket evolving along the ground state potential energy surface becomes more periodic in nature. Due to different compositions of the binary mixtures, two cosine functions were used to fit the coherent oscillation dynamics. The ground state recovery time (*τ*_3_) becomes smaller for neat-MeOH and neat-CHCl_3_ as compared to that of the binary mixtures. This indicates that the microheterogeneous environment has a good impact on the ground state recovery.

## Author Contributions

D.K.D. and D.G. wrote the main manuscript; D.K.D., K.M. and S.N.B. carried out the main laboratory experiments with advice from D.G. All authors reviewed the manuscript.

## Supplementary Material

Supplementary Information Supporting information

## Figures and Tables

**Table 1:** Ground state decay time constants of the binary mixture of MeOH-CHCl_3_ in IR775

% MeOH	*τ*_1_ (fs)	*τ*_3_ (ps)	*τ*_2_ (fs)	T_1_ (fs)	T_2_ (fs)	A_1_	A_2_
100	101	237	201	--	--	--	--
90	102	253	210	251	202	0.014	0.015
80	101	271	219	270	205	0.045	0.052
70	97	245	215	270	281	0.077	0.066
60	101	272	219	261	270	0.088	0.07
50	101	302	222	262	270	0.089	0.082
40	102	274	227	264	271	0.099	0.092
30	100	265	230	265	277	0.110	0.093
20	100	283	300	260	282	0.151	0.148
10	100	278	222	260	280	0.149	0.130
0	100	249	205	--	--	--	--
