# Supplementary material for: Direct Observation of Coherent Oscillations in Solution due to Microheterogeneous Environment
Source: Sci Rep. 2014 Aug 18;4:6097. doi: 10.1038/srep06097 (PMC4135331; doi:10.1038/srep06097)
Supplement: Supplementary Information — Supporting information [file srep06097-s1.doc]

**Supporting Information**

**Direct Observation of Coherent Oscillations in Solution due to Microheterogeneous Environment**

Dipak Kumar Das, Krishnandu Makhal, Soumendra Nath Bondhopadhya, Debabrata Goswami^*^

*Department of Chemistry, Indian Institute of Technology Kanpur, Kanpur-208016, Uttar Pradesh, India*

*[E-mail-dgoswami@iitk.ac.in](mailto:E-mail-dgoswami@iitk.ac.in)

Figure S1 : (1) Absorption spectrum of IR775 in 100% methanol (black dotted line) and the thin lines are its Gaussian deconvolution plots showing the transitions from the zeroth vibrational level in the ground state to the *i*^th^ (where i= 0, 1, 2, 3) vibrational level in the excited state.

Figure S1: (2) Absorption spectrum of IR775 in 90% methanol and 10% CHCl_3_ (black dotted line) and the thin lines are its Gaussian deconvolution plots showing the transitions from the zeroth vibrational level in the ground state to the *i*^th^ (where i= 0, 1, 2, 3) vibrational level in the excited state.

Figure S1: (3) Absorption spectrum of IR775 in 80% methanol and 20% CHCl_3_ (black dotted line) and the thin lines are its Gaussian deconvolution plots showing the transitions from the zeroth vibrational level in the ground state to the *i*^th^ (where i= 0, 1, 2, 3) vibrational level in the excited state.

Figure S1: (4) Absorption spectrum of IR775 in 70% methanol and 30% CHCl_3_ (black dotted line) and the thin lines are its Gaussian deconvolution plots showing the transitions from the zeroth vibrational level in the ground state to the *i*^th^ (where i= 0, 1, 2, 3) vibrational level in the excited state.

Figure S1: (5) Absorption spectrum of IR775 in 60% methanol and 40% CHCl_3_ (black dotted line) and the thin lines are its Gaussian deconvolution plots showing the transitions from the zeroth vibrational level in the ground state to the *i*^th^ (where i= 0, 1, 2, 3) vibrational level in the excited state.

Figure S1: (6) Absorption spectrum of IR775 in 50% methanol and 50% CHCl_3_ (black dotted line) and the thin lines are its Gaussian deconvolution plots showing the transitions from the zeroth vibrational level in the ground state to the *i*^th^ (where i= 0, 1, 2, 3) vibrational level in the excited state.

Figure S1: (7) Absorption spectrum of IR775 in 40% methanol and 60% CHCl_3_ (black dotted line) and the thin lines are its Gaussian deconvolution plots showing the transitions from the zeroth vibrational level in the ground state to the *i*^th^ (where i= 0, 1, 2, 3) vibrational level in the excited state.

Figure S1: (8) Absorption spectrum of IR775 in 30% methanol and 70% CHCl_3_ (black dotted line) and the thin lines are its Gaussian deconvolution plots showing the transitions from the zeroth vibrational level in the ground state to the *i*^th^ (where i= 0, 1, 2, 3) vibrational level in the excited state.

Figure S1: (9) Absorption spectrum of IR775 in 20% methanol and 80% CHCl_3_ (black dotted line) and the thin lines are its Gaussian deconvolution plots showing the transitions from the zeroth vibrational level in the ground state to the *i*^th^ (where i= 0, 1, 2, 3) vibrational level in the excited state.

Figure S1: (10) Absorption spectrum of IR775 in 10% methanol and 90% CHCl_3_ (black dotted line) and the thin lines are its Gaussian deconvolution plots showing the transitions from the zeroth vibrational level in the ground state to the *i*^th^ (where i= 0, 1, 2, 3) vibrational level in the excited state.

Figure S1: (11) Absorption spectrum of IR775 in 100% CHCl_3_ (black dotted line) and the thin lines are its Gaussian deconvolution plots showing the transitions from the zeroth vibrational level in the ground state to the *i*^th^ (where i= 0, 1, 2, 3) vibrational level in the excited state.

Figure S2: Schematic representation of the displacement of the electronic potential minima of the excited state potential with respect to the ground one.
